# Comparative epidemiology of human metapneumovirus- and respiratory syncytial virus-associated hospitalizations in Guatemala

**DOI:** 10.1111/irv.12251

**Published:** 2014-04-25

**Authors:** John P McCracken, Wences Arvelo, José Ortíz, Lissette Reyes, Jennifer Gray, Alejandra Estevez, Oscar Castañeda, Gayle Langley, Kim A Lindblade

**Affiliations:** a Center for Health Studies, Universidad del Valle de GuatemalaGuatemala City, Guatemala; b Division of Global Disease Detection and Emergency Response, Center for Global Health, Centers for Disease Control and PreventionAtlanta, GA, USA; c Guatemalan Social Security InstituteGuatemala City, Guatemala; d Ministry of Public Health and Social WelfareCuilapa, Santa Rosa, Guatemala; e Division of Viral Diseases, National Center for Immunization and Respiratory Diseases, Centers for Disease Control and PreventionAtlanta, GA, USA

**Keywords:** Acute respiratory infection, human metapneumovirus, pneumonia, respiratory syncytial virus, surveillance

## Abstract

**Background:**

Human metapneumovirus (HMPV) is an important cause of acute respiratory infections (ARI), but little is known about how it compares with respiratory syncytial virus (RSV) in Central America.

**Objectives:**

In this study, we describe hospitalized cases of HMPV- and RSV-ARI in Guatemala.

**Methods:**

We conducted surveillance at three hospitals (November 2007–December 2012) and tested nasopharyngeal and oropharyngeal swab specimens for HMPV and RSV using real-time reverse transcription-polymerase chain reaction. We calculated incidence rates, and compared the epidemiology and outcomes of HMPV-positive versus RSV-positive and RSV-HMPV-negative cases.

**Results:**

We enrolled and tested specimens from 6288 ARI cases; 596 (9%) were HMPV-positive and 1485 (24%) were RSV-positive. We observed a seasonal pattern of RSV but not HMPV. The proportion HMPV-positive was low (3%) and RSV-positive high (41%) for age <1 month, whereas these proportions were similar (∼20%) by age 2 years. The annual incidence of hospitalized HMPV-ARI was 102/100 000 children aged <5 years [95% confidence interval (CI): 75–178], 2·6/100 000 persons aged 5–17 years (95%CI: 1·2–5·0), and 2·6/100 000 persons aged ≥18 years (95%CI: 1·5–4·9). Among children aged <5 years, HMPV-positive cases were less severe than HMPV-RSV-negative cases after adjustment for confounders [odds ratio (OR) for intensive care = 0·63, 95% CI 0·47–0·84]; OR for death = 0·46, 95% CI 0·23–0·92).

**Conclusions:**

Human metapneumovirus is a substantial contributor to ARI hospitalization in Guatemala, but HMPV hospitalizations are less frequent than RSV and, in young children, less severe than other etiologies. Preventive interventions should take into account the wide variation in incidence by age and unpredictable timing of incidence peaks.

## Background

Human metapneumovirus (HMPV), a virus with genetic, epidemiological, and clinical similarities to respiratory syncytial virus (RSV), was first identified in 2001 among children with acute respiratory infections (ARI) in the Netherlands.^1^ The prevalence of HMPV infection among ARI cases has been described in North America,^2^ South America,^3^ Asia,^4^ the Middle East,^5^ Africa,^6^ and Oceania.^7^ The first report of HMPV infections in Central America was of a pediatric case series (*n* = 9) in Costa Rica during 2008.^8^ A study among both outpatients (*n* = 1573) and inpatients (*n* = 183) with influenza-like illness in El Salvador, Honduras and Nicaragua from August 2006 to April 2009 detected only three HMPV infections using immunofluorescence.^9^ Schlaudecker *et al*.^10^ used a multiplex polymerase chain reaction test and found 28 (8%) cases positive for HMPV out of 345 ARI cases in children presenting to outpatient clinics in Honduras from February 2010 to June 2011.

Evidence from previous studies has been inconsistent regarding whether ARI cases that are HMPV-positive tend to be more or less severe than HMPV-negative cases. Studies have shown greater likelihood of a clinical pneumonia diagnosis and need for mechanical ventilation associated with HMPV compared with HMPV-negative cases,^11^,^12^ but another study showed that children with HMPV were less likely to require admission to an intensive care unit (ICU) than those with RSV or influenza A.^2^ Other studies comparing HMPV and RSV infections did not find any evidence of a difference in the probability of requiring mechanical ventilation, having longer hospital stay,^12^,^13^ or being admitted to the hospital or ICU.^14^ However, as these studies did not adjust for age, it is unclear to what extent these comparisons are biased by differences in the age distributions of the children infected with each virus.^15^

In this manuscript, we present estimates from Guatemala of population-based incidence rates of hospitalized cases of ARI that have HMPV infection (HMPV-ARI), and we use comparisons with cases RSV-positive and those negative for both HMPV and RSV (HMPV-RSV-negative) to highlight important features of the epidemiology of HMPV infection, particularly, seasonality, age distribution and disease severity.

## Methods

### Data collection

We have conducted ARI surveillance in hospitals in the Departments of Santa Rosa (National Hospital of Cuilapa) since November 2007, Quetzaltenango (Western Regional Hospital) since February 2009, and Guatemala (Guatemalan Social Security Institute, IGSS) from November 2009 to April 2011. We previously described the surveillance sites in more detail.^16^,^17^ This analysis focuses on cases as the start of surveillance until December 31, 2012.

Surveillance nurses searched the emergency departments and hospital wards daily for patients with respiratory admission diagnoses or chief complaints and screened them for eligibility. Eligibility criteria were as follows: (i) hospitalization; (ii) at least one sign of acute infection [temperature ≥38°C, temperature <35·5°C, abnormal white blood cell count (age <5 years and count <5550 or >15 000 cells/μl; ≥5 years and count <3000 or >11 000 cells/μl), or abnormal white blood cell differential]; and (iii) at least one of the following respiratory signs or symptoms: cough, tachypnea (age <2 months and ≥60 breaths/min; age 2–11 months and ≥50 breaths/min; age 1–4 years and ≥40 breaths/min; age ≥5 years and ≥20 breaths/min), sputum production, pleuritic chest pain, hemoptysis, difficulty breathing, shortness of breath, sore throat, or among children aged <2 years, pausing repeatedly while breastfeeding or drinking, nasal flaring, or noisy breathing. Since February 2011, we have also included all children aged <5 years meeting the World Health Organization pneumonia case definition: cough or difficulty breathing with tachypnea, chest indrawing, stridor, or a danger sign (inability to feed, loss of consciousness, lethargy, and convulsions).

Surveillance nurses recorded information from medical charts, interviewed patients or caregivers, and took nasopharyngeal (NP) and oropharyngeal (OP) swabs. The nurses measured blood oxygen saturation by pulse oximetry, and we defined hypoxemia as saturation <90% in Santa Rosa (elevation approximately 900 meters), <89% in Guatemala City (elevation approximately 1500 meters), and <88% in Quetzaltenango (elevation approximately 2300 meters). Digital photographs of chest radiographs, if ordered by attending physician, were sent to a panel of three radiologists trained to interpret images according to World Health Organization guidelines.^18^

Nasopharyngeal and OP swabs were combined into one tube with viral transport media and sent to the Universidad del Valle de Guatemala, where they were tested by real-time reverse transcription-polymerase chain reaction (rRT-PCR) assays for HMPV, RSV, influenza A and B viruses, parainfluenza virus types 1, 2, and 3, and adenovirus,^19^ using primer/probe reagents and protocols provided by the US CDC. Briefly, rRT-PCR assays were performed on an ABI7500 using the AgPath-ID One-Step kit (Applied Biosystems, Foster City, CA, USA). Each reaction mixture contained 1× RT-PCR buffer, 1× RT-PCR enzyme, 1× primer and probe mixture and 5 μl of nucleic acid extract in a total reaction volume of 25 μl. Thermocycling conditions were as follows: 45°C for 10 min, 95°C for 10 min, and 45 cycles of 95°C for 15 sec, and 55°C for 1 min. For all of the viruses tested, specimens with cycle threshold values <40 were considered positive.

### Data analysis

We classified the cases into three etiological groups for comparisons: HMPV-positive, RSV-positive, and HMPV-RSV-negative. HMPV-RSV co-infections were included in both HMPV-positive and RSV-positive groups for descriptive analyses. We compared demographic characteristics, clinical presentation, and severity of disease between these three groups of patients. We plotted the weekly counts of HMPV-positive and RSV-positive cases. To evaluate the shape of the associations between the probabilities of each viral infection and the age of the patients, allowing for nonlinear associations, we fit generalized additive models (GAMs) for the log odds of HMPV and RSV infections with penalized spline terms for age, using generalized cross-validation to determine the degrees of freedom and running separate models by virus and age group (<5 years and ≥5 years).^20^ To assess the fit of these models, we divided the children aged <5 years into age twentiles and persons aged ≥5 years into age deciles, calculated the observed proportions of ARI cases positive for each virus, and overlaid these points at the median age for the corresponding age twentile or decile on the penalized spine plots.

For incidence rate calculations, we included cases only from predefined catchment areas in the departments of Santa Rosa^21^ and Quetzaltenango (municipalities of Almolonga, Cantel, Concepción Chiquirichapa, La Esperanza, Olintepeque, San Juan Ostuncalco, Quetzaltenango, Salcajá, San Mateo, Zunil). Incidence was calculated for Santa Rosa for years 2008 to 2012 and Quetzaltenango for years 2009 to 2012. Population denominators were obtained from the 2002 census data and official projections [National Statistics Institute (INE), Guatemala]. We adjusted rates upwards to account for: (i) the proportion of eligible cases by age group and surveillance site for which specimens were not tested for HMPV; and (ii) the proportion of the target populations with hospitalized ARI who sought care at the surveillance hospitals, as assessed by healthcare utilization surveys (HUS). In the HUS, among those who reported seeking care at a hospital in the previous 12 months for cough and difficulty breathing for ≥2 days, or clinician-diagnosed pneumonia, 53% in Santa Rosa^21^ and 60% in Quetzaltenango^22^ sought care for their illness at surveillance hospitals. We estimated the uncertainty in the adjusted incidence rates by repeating these calculations 1999 times, each time: (i) sampling from the binomial distribution [∼Bin(*n* = number missing, *P* = probability of HMPV among those tested)] to impute the number of HMPV infections among the eligible cases not tested for HMPV; (ii) bootstrap sampling from the HUS dataset the proportion of ARI cases for which care was sought at surveillance hospitals; and (iii) sampling from the Poisson distribution based on adjusted counts. We used the bias corrected method to calculate 95% confidence intervals.^23^

We defined severe cases as those requiring intensive care, mechanical ventilation, or hospitalization >1 week, and those that resulted in death. We used logistic regression to test for association between etiological group (excluding HMPV-RSV co-infections) and the odds of these indicators of severity. Separate models were fit by age group (<5 and ≥5 years), and we present crude and adjusted associations, conditioning on age, sex, and surveillance area. As age ≥5 years is a broad age range across which to assume associations with disease severity are constant, we tested for interaction between etiological group and age (5–17, 18–49, and ≥50 years).

### Human subjects

The surveillance protocol was approved by the ethics review committee from the Universidad del Valle de Guatemala (Guatemala City, Guatemala) and the US Centers for Disease Control and Prevention (Atlanta, GA, USA), and by the MSPAS (Guatemala City, Guatemala). Eligible patients who provided written, informed consent were enrolled in the study. For patients aged <18 years, parents or guardians served as proxies, and children aged 7–17 years were asked to provide written, informed assent.

## Results

We identified 7289 eligible ARI cases, 6597 (91%) were enrolled, and 6288 (95%) of those enrolled had a specimen tested. Of these, rRT-PCR assays detected HMPV in 596 (9%) and RSV in 1485 (24%) cases; 43 (0·7%) HMPV-RSV co-infections were identified. Of the HMPV-positive cases, 87% occurred in children aged <5 years, similar to proportion of the RSV-positive cases (91%), whereas only about half of the HMPV-RSV-negative cases occurred in this age group (Table1).

**Table 1:** Demographic characteristics, clinical presentation, and hospital course of patients by etiological classification and age group at three Guatemalan hospitals, November 2007–August 2012

	HMPV-positive (*n* = 596)*	RSV-positive (*n* = 1485)*	HMPV-RSV-negative cases (*n* = 4248)
Age < 5 years	520 (87)	1356 (91)	2295 (54)
Age, year (med [IQR])	1·0 [0·5, 1·7]	0·5 [0·2, 1·0]	0·8 [0·3, 1·7]
Sex (female)	222 (43)	591 (44)	960 (42)
Cough	513 (99)	1330 (98)	2081 (92)
Temperature (med [IQR])	38·0 [37·4, 38·5]	38·0 [37·0, 38·5]	37·8 [37·0, 38·5]
Difficulty breathing	440 (85)	1162 (86)	1814 (80)
Tachypnea	238 (46)	556 (41)	880 (38)
Chest indrawing	176/472 (37)	690/1294 (53)	843/1978 (43)
Stridor	24/472 (5)	71/1295 (5)	144/1982 (7)
Hypoxemia	141/447 (32)	440/1245 (35)	497/1891 (26)
Radiological pneumonia	84/293 (29)	228/707 (32)	325/1190 (27)
Hyperaeration	147/304 (48)	410/740 (55)	526/1250 (42)
Discharge Dx pneumonia	402 (79)	1042 (77)	1550 (69)
Discharge Dx bronchitis	136 (27)	300 (23)	424 (19)
Intensive care admission	67 (13)	252 (19)	489 (21)
Mechanical ventilation	16 (3)	87 (6)	198 (9)
Hospitalized >1 week	133/508 (26)	427/1353 (32)	787/2262 (35)
Death	9/508 (2)	35/1353 (3)	101/2263 (4)
Age ≥ 5 years	76 (13)	129 (9)	1953 (46)
Age, year (med [IQR])	47 [10,66]	50 [20, 68]	50 [26, 69]
Sex (female)	42 (55)	69 (53)	1013 (52)
Cough	69 (91)	111 (89)	1693 (91)
Temperature (med [IQR])	38·0 [37·0, 38·4]	37·5 [37·0, 38·0]	37·5 [37·0, 38·0]
Difficulty breathing	65 (86)	95 (76)	1508 (81)
Hypoxemia	31/66 (47)	49/110 (45)	718/1641 (44)
Radiological pneumonia	25/48 (52)	34/71 (48)	507/991 (51)
Hyperaeration	21/48 (44)	36/76 (47)	471/1038 (45)
Discharge Dx pneumonia	27 (36)	45 (35)	705 (37)
Discharge Dx bronchitis	1 (1)	0	9 (0)
Intensive care admission	8 (11)	14 (11)	224 (11)
Mechanical ventilation	4 (5)	13 (10)	158 (8)
Hospitalized >1 week	34/74 (46)	64/127 (50)	896/1919 (47)
Death	8/74 (11)	16/127 (13)	204/1925 (11)

* Includes 43 cases with HMPV-RSV co-infections.

Of the 2295 HMPV-RSV-negative cases in children aged <5 years, we detected the following viruses: 180 (8%) influenza A, 43 (2%) influenza B, 99 (5%) parainfluenza virus type 1, 28 (1%) parainfluenza virus type 2, 251 (12%) parainfluenza virus type 3, and 293 (14%) adenovirus. Of the 1953 HMPV-RSV-negative cases in persons aged ≥5 years, we detected the following viruses: 153 (8%) influenza A, 30 (2%) influenza B, 28 (1%) parainfluenza virus 1, 10 (1%) parainfluenza virus 2, 62 (3%) parainfluenza virus 3, and 118 (6%) adenovirus. At least one other virus was also detected in 127 (24%) of the HMPV-positive cases in children aged <5 years and in 13 (17%) of the HMPV-positive cases among persons aged ≥5 years. At least one other virus was also detected in 271 (20%) of the RSV-positive cases in children aged <5 years and in 33 (26%) of the RSV-positive cases among persons aged ≥5 years.

Among children aged <5 years with ARI, the median age was older for those HMPV-positive (1 year) compared with those RSV-positive (6 months). There was no major difference in the respiratory signs and symptoms, presence of fever, or radiological diagnoses between the three etiological groups presented in Table1. Among children aged <5 years, hospital course (intensive care, mechanical ventilation, length of stay) and mortality proportions suggest that severity is lowest for HMPV-positive, intermediate for RSV-positive, and greatest for HMPV-RSV-negative. Among those aged ≥5 years, there was no consistent association between these indicators of severity and etiological group.

**Figure 1:**
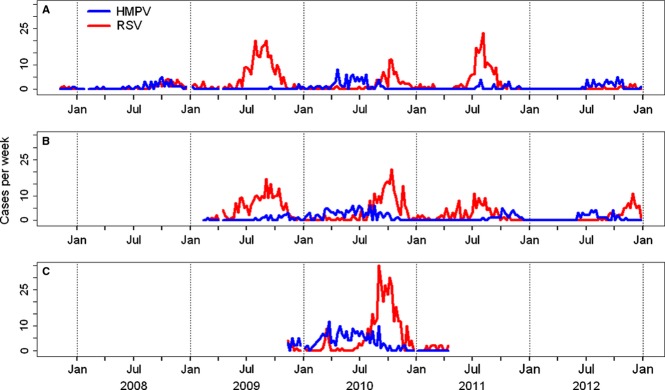
A–C. Weekly numbers of human metapneumovirus (HMPV) and respiratory syncytial virus (RSV) infections among persons hospitalized with acute respiratory infections in Guatemala: (A) Santa Rosa, (B) Quetzaltenango, and (C) Guatemala City.

In Santa Rosa during the years 2008, 2009, 2010, 2011, and 2012, we detected HMPV in 42 (14%), 5 (1%), 75 (15%), 16 (3%), and 43 (10%) of ARI cases and RSV in 33 (11%), 242 (38%), 81 (17%), 161 (33%), and 9 (2%) of ARI cases, respectively. In Quetzaltenango during the years 2009, 2010, 2011, and 2012, we detected HMPV infection in 21 (3%), 111 (13%), 31 (7%), and 37 (8%) of ARI cases and RSV in 223 (34%), 195 (22%), 118 (26%), and 69 (15%) of ARI cases, respectively. In Guatemala City during 2010, we detected HMPV infection in 195 (16%) of ARI cases and RSV in 330 (27%). RSV infections mostly occurred from August to October, during the second half of the rainy season, with the exception of 2012, when there were few RSV cases and most occurred during November–December. In contrast, HMPV peaked at different times each year (Figure1).

**Figure 2:**
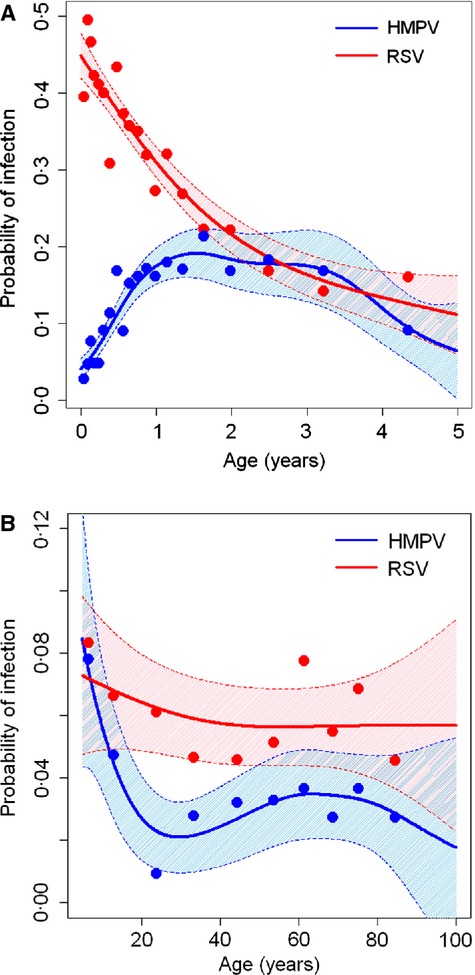
A–B. Relationships between age and probabilities of HMPV and RSV among persons aged (A) <5 years and (B) ≥5 years hospitalized with acute respiratory infection; line estimated using penalized spines in generalized additive models, shaded areas indicate 95% confidence intervals, and points indicate observed proportions and median age within twentiles of age for children aged <5 years (A) and deciles of age for persons aged ≥5 years (B).

Among children aged <5 years, statistically significant nonlinear relationships with age were found for both HMPV [degrees of freedom (d.f.) = 5·2, *P*-value <0·001) and RSV (d.f. = 2·1, *P*-value <0·001). The probability of HMPV was low during the first month of life and increased with age, peaking at 20% around age 16 months. The predicted probability of RSV was approximately 41% during the first month of life, after which it declined rapidly, becoming similar to the probability of HMPV by around age 2 years (Figure2). Among persons aged ≥5 years, the probability of HMPV declined from 8% at age 5 years to around 2·5% at age 20 years (d.f. = 3·6, *P*-value = 0·005). In contrast, there was no statistically significant evidence of an association between age and the probability of RSV in this age group (*P*-value = 0.402).

The crude annual incidence rates of hospitalized HMPV-ARI (Table3) were 56 cases per 100 000 children aged <5 years (95% CI: 49, 64), 1·4 cases per 100 000 children aged 5–17 years (95% CI: 0·8, 2·4), and 1·5 cases per 100 000 persons aged ≥18 years (95% CI: 0·9, 2·2). The estimated annual incidence rates adjusted for the proportion of eligible cases tested for HMPV and for proportions of the populations seeking health care at the surveillance hospitals were 102 cases per 100 000 children aged <5 years (95% CI: 75, 178), 2·6 cases per 100 000 children aged 5–17 years (95% CI: 1·2, 5·0), and 2·6 cases per 100 000 persons aged ≥18 years (95% CI: 1·5, 4·9).

In unadjusted logistic regression models (Table3), among children aged <5 years, having HMPV infection compared with RSV infection was associated with significantly lower odds of being admitted to the ICU, needing mechanical ventilation and being hospitalized ≥1 week. After adjusting these models for age, sex and surveillance site, there was no statistically significant association. Among children aged <5 years, after adjustment for potential confounders, we found that HMPV-positive cases versus HMPV-RSV-negative cases were associated with lower odds of ICU admission (*P*-value = 0·002), mechanical ventilation (*P*-value<0·001), hospital stay >1 week (*P*-value = 0·011), and death (*P*-value = 0·026). Among persons aged ≥5 years, there was no evidence of a difference in severity associated with etiological group, and we did not find any evidence of effect modification by age (Table3).

**Table 2:** Annual crude and adjusted incidence rates (95% confidence intervals) of hospitalized acute respiratory infections with HMPV by age group—Santa Rosa, 2008–2012 and Quetzaltenango, 2009–2012

			Rates per 100 000 persons per year		
			
	HMPV cases	Person-years	Crude	Adjustment for enrollment and RSV testing^1^	Plus adjustment for healthcare seeking^1^^,^^2^
All ages	265	27 749 038	9·5 (8·4, 10·8)	9·9 (8·3, 10·9)	17·4 (11·9, 25·0)
<5 years	231	412 560	56·0 (49·0, 63·7)	57·7 (49·9, 64·5)	101·6 (75·0, 177·6)
<6 months	48	42 456	113·1 (83·4, 149·9)	114·8 (82·4, 148·4)	205·2 (133·5, 385·1)
6–23 months	148	125 880	117·6 (99·4, 138·1)	123·1 (103·3, 143·0)	215·3 (155·3, 386·6)
2–4 years	35	244 224	14·3 (10·0, 19·9)	14·5 (9·8, 19·7)	25·3 (15·7, 45·5)
5–17 years	13	921 286	1·4 (0·8, 2·4)	1·5 (0·8, 2·4)	2·6 (1·2, 5·0)
≥18 years	21	1 441 092	1·5 (0·9, 2·2)	1·5 (0·9, 2·2)	2·6 (1·5, 4·9)
18–49 years	5	1 089 578	0·5 (0·1, 1·1)	0·5 (0·2, 1·0)	0·8 (0·2, 2·0)
≥50 years	16	351 514	4·6 (2·6, 7·4)	4·7 (2·6, 7·1)	8·4 (4·4, 16·7)
50–64 years	6	212 973	2·8 (1·0, 6·1)	3·0 (0·9, 6·1)	5·4 (1·8, 12·7)
≥65 years	10	138 541	7·2 (3·5, 13·3)	7·3 (3·6, 13·0)	13·0 (5·4, 28·0)

1 Adjusted for proportion of eligible cases enrolled and tested for HMPV by age group and site, which ranged from 90% to 100%.

2 Adjusted for the proportion in health utilization surveys stating that they were hospitalized at the surveillance hospital among those hospitalized for pneumonia from Santa Rosa (53%) and from Quetzaltenango (60%).

**Table 3:** Odds ratios (95% confidence intervals) for indicators of disease severity associated with HMPV among patients hospitalized with acute respiratory infection

Outcome	HMPV-positive versus RSV-positive*		HMPV-positive versus HMPV-RSV-negative*	
	
	Crude	Adjusted**	Crude	Adjusted**
Age < 5 years	(*n* = 1804)***		(*n* = 2815)***	
Intensive care	0·66 (0·49, 0·89)	1·08 (0·78, 1·50)	0·55 (0·41, 0·72)	0·63 (0·47, 0·84)
Mechanically ventilated	0·46 (0·26, 0·80)	0·70 (0·38, 1·27)	0·34 (0·20, 0·56)	0·38 (0·23, 0·65)
Hospitalized > 1 week	0·78 (0·62, 0·99)	1·24 (0·96, 1·61)	0·66 (0·54, 0·82)	0·75 (0·60, 0·93)
Death	0·65 (0·30, 1·41)	0·75 (0·33, 1·71)	0·39 (0·19, 0·77)	0·46 (0·23, 0·92)
Age ≥5 years	(*n* = 191)***		(*n* = 2029)***	
Intensive care	1·01 (0·40, 2·55)	0·94 (0·34, 2·64)	0·91 (0·43, 1·91)	0·78 (0·37, 1·67)
Mechanically ventilated	0·52 (0·16, 1·65)	0·71 (0·20, 2·47)	0·63 (0·23, 1·75)	0·79 (0·28, 2·22)
Hospitalized > 1 week	0·86 (0·47, 1·56)	1·06 (0·55, 2·04)	0·97 (0·61, 1·55)	1·14 (0·70, 1·85)
Death	0·88 (0·36, 2·18)	1·04 (0·38, 2·86)	1·02 (0·48, 2·16)	1·31 (0·61, 2·84)

* Cases with HMPV-RSV co-infection (*n* = 43) excluded from these models.

** OR estimates adjusted for age (cubic spline with 9 degrees of freedom), sex, and surveillance area (Santa Rosa, Quetzaltenango, Guatemala).

*** Sample sizes for some models were lower by about 1–2% due to missing data for duration of hospitalization and death.

## Discussion

To our knowledge, this is the first report from Central America of HMPV infection in the context of systematic hospital-based surveillance for ARI, and these data from three sites in Guatemala covering a span of 5 years demonstrate that HMPV is a substantial cause of ARI hospitalization in Guatemala. Among children aged <5 years, we found population incidence rates of HMPV-positive ARI similar to a previous study among US children.^24^ In contrast, the incidence rate we found for adults was many times lower than that reported in Tennessee, USA.^25^ However, lower access to and utilization of healthcare services may result in underestimation of the burden in Guatemala, despite our attempt to correct for healthcare seeking behavior. The proportion of ARI cases HMPV-positive among children aged <5 years in our study (13%) was higher than other previous studies from USA (New York and Tennessee), Italy, France and Korea,^26^–^29^ but similar to studies in Alaska and Brazil.^12^,^30^ The proportion of hospitalized ARI cases that were HMPV-positive among adults ≥50 years (3%) was similar to that reported in TN, USA (4·5%).^25^

There was no clear seasonality of HMPV infection that we observed. In contrast, it has been reported that peak incidence of HMPV coincides with the latter half of RSV seasonal peaks in both the USA and Brazil.^31^,^32^ The broad distribution of HMPV cases over time, in contrast to the sharper peak of RSV, is, however, consistent with previous studies in both temperate and tropical environments.^31^–^33^ The wide variation we found between years in the numbers and proportions of ARI cases attributable to HMPV infection is also consistent with findings from prospective ARI surveillance in other countries.^31^,^32^

Our study is consistent with previous findings that HMPV and RSV are major causes of ARI during early childhood and fairly important during later childhood and adulthood, and that HMPV-ARI is less frequent than RSV-ARI.^34^ Hall *et al*. 35found that incidence of RSV hospitalization in the United States was highest for infants 1 month of age, which is similar to our findings for proportion of ARI cases RSV-positive. Our detailed analysis of the association between age and the probability of detecting each virus among ARI cases, however, provides new information about the relative burden of disease attributable to each virus at specific ages. For instance, during the neonatal period, HMPV was detected in only about 3% of ARI cases, whereas RSV was detected in 41%. Most population-based studies that have reported the proportions of ARI positive for HMPV and RSV have not provided this level of detail because they have grouped children aged <6 months or <2 years.^27^,^36^,^37^ The decline in RSV and increase in HMPV over the first year of life suggest that the timing of vaccination would have important implications for the relative burden of disease that would be prevented by potential future HMPV and RSV vaccines. The small number of HMPV cases in the first months of life suggests that an effective early childhood vaccine could prevent the majority of its disease burden, in contrast to RSV, which may require maternal immunization to prevent the high burden during the first few months of life.

Among children aged <5 years, after adjusting for potential confounders, ARI cases with HMPV were no less severe than those with RSV but were less severe than those negative for both viruses, which included 1512 (66%) negative for the eight viruses tested (data not shown). Among persons aged ≥5 years, there was no evidence of a difference in severity when comparing HMPV-positive to either RSV-positive or HMPV-RSV-negative cases, which included 1575 (81%) negative for the eight viruses tested. Our crude results are consistent with evidence that HMPV hospitalizations are less severe and of shorter duration compared with RSV hospitalizations.^32^,^38^ However, it is also informative to know, as the adjusted models indicate, that children aged <5 years of similar age and same sex admitted to the same hospital would be expected to have similar hospital course and outcomes, regardless of whether the infection is HMPV or RSV, but that HMPV-positive cases can be expected to be less severe than HMPV-RSV-negative cases.

Our study has a few important limitations. Our initial ARI case definition is likely to have missed a proportion of illnesses caused by HMPV and RSV, particularly among young infants and adults, as they are often afebrile and lack significant WBC abnormalities.^39^–^41^ However, this underestimation of burden and potential selection bias was no longer a limitation for children aged <5 years after we added the WHO pneumonia case definition in February 2011. Furthermore, the small proportion (9%) of cases after February 2011 in children aged <5 years that lacked one of these signs of infection suggests this source of bias was not substantial for this age group. Interpretation of the comparisons between HMPV and HMPV-RSV-negative cases is limited by the incomplete information on the etiologies of this heterogeneous reference group, among which we detected a viral infection in only 27% of the cases, as well as the diverse clinical and epidemiological features of these infections. Our crude population incidence rates of HMPV-ARI hospitalization are known to substantially underestimate the burden of severe disease in the community that should have led to hospitalization. On the other hand, the adjustment of incidence rates for healthcare seeking behavior was based on self-reported illness and healthcare seeking practices, which introduces substantial uncertainty due to small sample size, as reflected in the wider confidence intervals, and is also subject to reporting bias.

Our study suggests that HMPV is important though less frequent than RSV as a cause of ARI hospitalizations and mortality in Guatemala. These findings highlight the importance of considering HMPV testing within ARI surveillance platforms in Central America. Additionally, interventions to prevent ARI due to HMPV and evaluations of their impact should take into account the potentially unpredictable timing of incidence peaks, rarity of cases during the first few months of life, and lesser severity compared with HMPV-RSV-negative cases among young children.
